# *In Vitro* Antioxidant *versus* Metal Ion Chelating Properties of Flavonoids: A Structure-Activity Investigation

**DOI:** 10.1371/journal.pone.0165575

**Published:** 2016-10-27

**Authors:** Sabri Ahmed Cherrak, Nassima Mokhtari-Soulimane, Farid Berroukeche, Bachir Bensenane, Angéline Cherbonnel, Hafida Merzouk, Mourad Elhabiri

**Affiliations:** 1 Laboratory of Physiology, Pathophysiology and Biochemistry of Nutrition, Department of Biology, Faculty of Natural and Life Sciences, Earth and Universe, Abou Bekr Belkaid University, 13000 Tlemcen, Algeria; 2 Laboratory of Bioorganic and Medicinal Chemistry, UMR 7509 CNRS, ECPM, 25 rue Becquerel, 67200 Strasbourg, France; Islamic Azad University Mashhad Branch, ISLAMIC REPUBLIC OF IRAN

## Abstract

Natural flavonoids such as quercetin, (+)catechin and rutin as well as four methoxylated derivatives of quercetin used as models were investigated to elucidate their impact on the oxidant and antioxidant status of human red blood cells (RBCs). The impact of these compounds against metal toxicity was studied as well as their antiradical activities with DPPH assay. Antihemolytic experiments were conducted on quercetin, (+)catechin and rutin with excess of Fe, Cu and Zn (400 μM), and the oxidant (malondialdehyde, carbonyl proteins) and antioxidant (reduced glutathione, catalase activity) markers were evaluated. The results showed that Fe and Zn have the highest prooxidant effect (37 and 33% of hemolysis, respectively). Quercetin, rutin and (+)catechin exhibited strong antioxidant properties toward Fe, but this effect was decreased with respect to Zn ions. However, the Cu showed a weak antioxidant effect at the highest flavonoid concentration (200 μM), while a prooxidant effect was observed at the lowest flavonoid concentration (100 μM). These results are in agreement with the physico-chemical and antiradical data which demonstrated that binding of the metal ions (for FeNTA: (+)Catechin, *K*_LFeNTA_ = 1.6(1) × 10^6^ M^-1^ > Rutin, *K*_LFeNTA_ = 2.0(9) × 10^5^ M^-1^ > Quercetin, *K*_LFeNTA_ = 1.0(7) × 10^5^ M^-1^ > Q35OH, *K*_LFeNTA_ = 6.3(8.7) × 10^4^ M^-1^ > Quercetin3’4’OH and Quercetin 3OH, *K*_LFeNTA_ ~ 2 × 10^4^ M^-1^) reflects the (anti)oxidant status of the RBCs. This study reveals that flavonoids have both prooxidant and antioxidant activity depending on the nature and concentration of the flavonoids and metal ions.

## Introduction

Flavonoids that belong to the polyphenols family are secondary plant metabolites and one the most occurring groups of phytochemicals. They occur in fruits, seeds, flowers and vegetables among others and are of important in the human diet. These compounds are of high physiological and morphological importance in plants [1, 2]. Protection of the plant from UV radiation, pigmentation in fruits and flowers or signalling properties (allelochemicals) can be cited. Flavonoids have an important function in plant defence. They can be transferred to the soil and inhibit the growth of the competitors. They can also act as signalling compounds for microbes or bacteria [3, 4]. Many flavonoids have also antifungal [5] and insecticide properties [6]. Last but not least, these phenolic compounds mainly regulate the antioxidant balance of the plants and prevent the plant from mutagenesis.

In humans, consumption of plant polyphenolic compounds (from fruit, vegetables, tea, red wine…) was also shown to contribute to numerous health benefits (pleiotropic action) [7]. Polyphenols display a broad spectrum of physiological activities such as anti-allergenic, anti-atherogenic, antimicrobial or cardioprotective effects, to cite a few [8]. For instance, polyphenols-rich diets have been associated with a lower risk of cancer and coronary heart disease mortality in a number of epidemiological studies [9, 10]. These beneficial effects were proposed to be related to reduced exposure to oxidative stress [11, 12].

**Fig 1:**
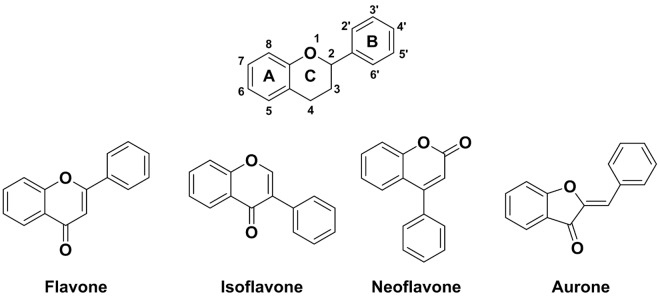
Basic structure of flavonoids and general chemical structure of different flavonoids.

Even though flavonoids share a common C_6_-C_3_-C_6_ (benzo-γ-pyrone) carbon skeleton (**Fig 1**), a wide range of structural diversity exists. They are usually found with various degrees of hydroxylation, methoxylation, glycosylation or glucuronidation [13, 14], which contributes to the great variety in biological properties and form the largest group in the polyphenol family with over 6,000 compounds identified so far [13].

The main biological activity of flavonoids thoroughly studied so far is their antioxidant activity. During normal metabolism, reactive oxygen species (ROS/RNS, e.g. hydroxyl OH^•^, superoxide O_2_^•^ˉ, nitric oxide NO^•^, nitrogen dioxide NO_2_^•^, peroxyl ROO^•^ and lipid peroxyl LOO^•^ to cite a few) are constantly formed and are believed to contribute to cellular aging, [15, 16] mutagenesis or carcinogenesis [17]. It is indeed thought that ROS produce these effects by DNA damages or LDL oxidation [18]. Flavonoids have been demonstrated to be essential radical scavengers [19, 20] because of their ability to stabilize free radicals and other active species. These antiradical/antioxidant capacities are intimately related to the redox properties of their phenolic hydroxyl groups, which can be easily oxidized [21]. As a consequence, their conjugated rings and hydroxyl groups allow them to act as radical scavengers, reducing the effect of ROS in the body. Structure-reactivity studies have demonstrated that the antiradical/antioxidant activities are related to structural criteria such as: (*i*) presence of an ortho-hydroxyl on the B-ring, (*ii*) presence of one or several free hydroxyl groups, (*iii*) presence of a C2-C3 double bond in the C-ring, or (*iv*) presence of a 3-hydroxyl group (**Fig 1**) [22–24]. Another potential mechanism by which flavonoids act as antioxidants relates to their interactions with redox enzymes. Flavonoids were shown to activate detoxifying enzymes such as NAD(P)H-quinone oxidoreductase, glutathione S-transferase or UDP-glucuronosyl transferase, which all belong to the defence arsenal towards oxidative stress [25].

The ability of flavonoids to prevent or scavenge the toxicity of redox active metal ions such as Fe or Cu has been less considered. These cations are believed to catalyze the production of oxidant species leading to lipid peroxidation, DNA and protein damages [26, 27]. Fe(II), even in tiny amounts, catalyzes, in the presence of hydrogen peroxide, the formation of hydroxyl radical ^•^OH by the well known redox cycling process known as Fenton cycle [28, 29]. Cu displays a more intricate behaviour [30]. Reaction of Cu(II) with H_2_O_2_ can occur via a “free radical” mechanism in which Cu(II) oxidizes H_2_O_2_ to O_2_^•^ with Cu(I) being formed and reacting with excess H_2_O_2_ to form HO^•^ (Fenton-like reaction). It was also suggested that rather than changing its oxidation state, Cu(II) forms a complex with peroxide with no formation of radical species (*i*.*e*., “complex” mechanism) [31]. Other studies [30] suggested that a higher oxidation state of copper (*i*.*e*., Cu(III) formed from Cu(I)+H_2_O_2_ + 2H^+^ ⇿Cu(III) + 2H_2_O) may be a reactive oxidizing intermediate that reacted with various substrates at rate constants that are, however, several orders of magnitude lower than the corresponding reactions with ^•^OH. Given these facts, the ability of flavonoids to strongly chelate prooxidant metal ions such as Cu or Fe that are involved in oxidants/radicals formation contributes as well to their antioxidant properties. A large number of plant flavonoids are capable to form stable metal complexes through their multiple OH groups and the carbonyl moiety, whenever present [32]. For instance, quercetin, that is characterized by three potential bidentate binding sites (α-hydroxy-carbonyl, β-hydroxy-carbonyl or catechol), can lead to stable metallic complexes [33]. Complexation of metal cations by quercetin has been already reported for a large number of metal ions such as Mo(VI), Fe(II)/Fe(III), Cu(II), Zn(II), Al(III), Tb(III), Pb(II), Co(II) [34, 35].

**Fig 2:**
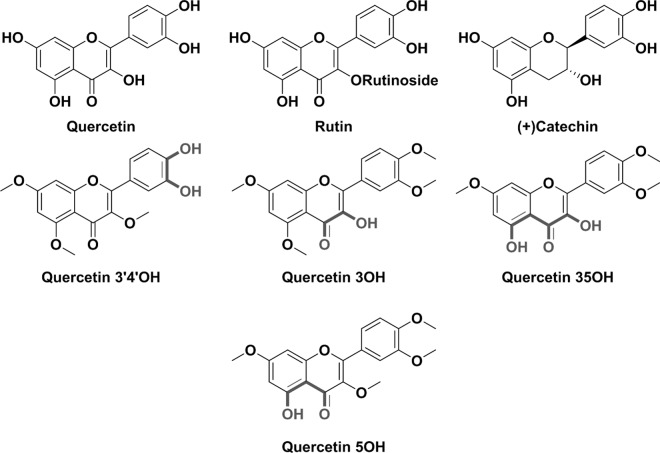
Chemical structures of quercetin, rutin, (+)-catechin and of the four polymethylated analogues of quercetin (the thick grey colour points out the potential bidentate binding sites).

Red blood cells (RBCs) membrane is the widest and most used model for studying biomembrane oxidative damage [36]. As most of the membranes, it plays a fundamental role in maintaining cellular homeostasis of RBCs. RBCs are indeed submitted to continuous fluxes of oxidative stress during their normal aerobic functions. But, in healthy subjects, this stress is balanced out by a powerful and efficient enzymatic and non enzymatic antioxidant network [37]. RBCs are especially susceptible to oxidation due to their high content of polyunsaturated lipids, their rich oxygen supply and the presence of transition metals such as Fe and Cu[38]. The aim of the present study was therefore to investigate, using physico-chemical tools, the metal binding capacities of selected flavonoids of interest that are commonly found in the human diet (*e*.*g*., quercetin, rutin and (+)catechin) as well as synthetic models of quercetin (*i*.*e*., four polymethylated derivatives of quercetin) that retain one or two bidentate binding sites (**Fig 2**). The antiradical properties (as scavengers of the 2,2-diphenyl-1-picrylhydrazyl DPPH radical) of these latter natural or synthetic flavonoids were then evaluated *in vitro*. Finally, the resistance of human RBCs to metal-induced hemolysis in the absence or in the presence of flavonoids was assessed *in vitro* as well as their oxidant/antioxidant status. This study was not devoted to mimic the *in vivo* conditions since the bioavailability of flavonoids is rather low [39, 40] and they would be found in low concentrations in human plasma. This fundamental approach allowed highlighting relationships between the chemical structure of the flavonoids and their effectiveness and ability to complex metal transition ions and to prevent RBCs hemolysis.

## Methods

### Solvent and Materials for the Physico-chemistry Investigations

(+)-Catechin (C_15_H_14_O_6_, MW = 290.27 g mol^-1^) was extracted from green tea by the group of Dr. A. P. Davies (Unilever, Bedford, UK) and was used without further purification[41]. Quercetin dihydrate (C_15_H_10_O_7_.2H_2_O, MW = 338.27 g mol^-1^, Sigma-Aldrich, 98%) and rutin trihydrate (C_27_H_30_O_16_.3H_2_O, MW = 664.56 g mol^-1^, Sigma-Aldrich, 95%) were purchased from Sigma-Aldrich and were used without further purification. NTA (nitrilotriacetate trisodium salt, Fluka, purum) was used as received. Quercetin derivatives such as 3,5,7-tri-O-methyl-quercetin (noted hereafter quercetin-3’4’OH), 5,7,3’,4’-tetra-O-methyl-quercetin (noted hereafter quercetin-3OH), 7,3’,4’-tri-O-methyl-quercetin (noted hereafter quercetin-35OH) and 3,7,3’,4’-tetra-O-methyl-quercetin (noted hereafter quercetin-5OH) were prepared according literature procedures [42–44].

For the sake of solubility, the investigated flavonoids and models were dissolved in a mixed solvent made of 80% of methanol (Merck) and 20% of water by weight. Distilled water was purified by passing it through a mixed bed of ion-exchanger (Bioblock Scientific R3-83002, M3-83006) and activated carbon (Bioblock Scientific ORC-83005) and was de-oxygenated by CO_2_- and O_2_-free argon (Sigma Oxiclear cartridge) before use. Spectrophotometric grade methanol (Merck, p.a.) was also de-oxygenated by CO_2_- and O_2_-free argon (Sigma Oxiclear cartridge). All the stock solutions were prepared by weighing solid products using an AG 245 Mettler Toledo analytical balance (precision 0.01 mg).

Copper(II) perchlorate hexahydrate (Cu(ClO_4_)_2_•6H_2_O, MW = 370.54 g mol^-1^, reagent grade), Zinc(II) perchlorate hexahydrate (Zn(ClO_4_)_2_•6H_2_O, MW = 327.38 g mol^-1^, reagent grade) and Fe(III) perchlorate hydrate (Fe(ClO_4_)_3_•xH_2_O, MW = 354.20 g mol^-1^ anhydrous basis, reagent grade) were purchased from Alfa Aesar and their stock solutions (~ 5–8 × 10^−2^ M) were prepared from their solid salts in water saturated with argon. The metal contents of the solutions were determined according to the classical colorimetric titrations [45]. The cupric solutions (Cu(ClO_4_)_2_×6H_2_O) were acidified with 0.1 M HClO_4_ to avoid hydroxide precipitation and their concentrations were ascertained by colorimetric titrations with standardized Na_2_H_2_EDTA solution (Merck, Titriplex III, 0.1 M) using ammonium acetate (Prolabo, Rectapur) as buffer and PAR (4-2(2-Pyridylazo)resorcinol monosodium salt monohydrate) as indicator. The concentrations of the Zn(II) solutions (Zn(ClO_4_)_2_×6H_2_O) stock solutions were ascertained by colorimetric titrations with Na_2_H_2_EDTA solution (Merck, Titriplex III, 0.1 M), ammoniac (Prolabo, Rectapur) and buffer tablet indicator (Merck). The concentration of Fe(III) stock solutions was ascertained by UV-Vis. absorption spectrophotometry (ε^240^ = 4.16×10^3^ M^-1^ cm^-1^ and ε^260^ = 2.88 × 10^3^ M^-1^ cm^-1^ in diluted aqueous perchloric acid solution; 4% *v/v* of HClO_4_ at 70%) [46].

*CAUTION! Perchlorate salts combined with organic ligands are potentially explosive and should be handled in small quantities and with the adequate precautions* [47].

The Fe**NTA** stock solutions (~10^−3^ M) were prepared by mixing equimolar amounts of Fe(III) perchlorate and NTA. A little excess of NTA was used to ensure complete Fe(III) binding and avoid formation of insoluble ferric tris(hydroxide) at pH 7.4. A Hepes buffer solution (0.1 M) was prepared at pH 7.4 in methanol/water mixture (80/20 w/w).

### Absorption spectrophotometric Titrations of the Polyphenols by FeNTA, Cu(II) and Zn(II) at pH 7.4

Microvolumes of concentrated Fe**NTA**, Cu(II) or Zn(II) salts were added to 2 mL of the flavonoid solution (2–5 × 10^−5^ M) in a 1 cm path length optical Hellma cell. The corresponding UV-Vis. spectra were recorded from 230 nm to 800 nm to follow the evolution of complexation on a Cary 50 (Varian) or a Cary 5000 (Agilent) spectrophotometer maintained at (25.0 ± 2°C. The spectrophotometric data were then analyzed with Specfit [48] program which adjusts the absorptivities and the stability constants of the species formed at equilibrium. Specfit uses factor analysis to reduce the absorbance matrix and to extract the eigenvalues prior to the multiwavelength fit of the reduced data set according to the Marquardt algorithm[49, 50].

### Antioxidant Activities Assessed with DPPH Radicals

The antioxidant/antiradical properties of the flavonoids were estimated by the radical scavenging activity method using 2,2-diphenyl-1-picrylhydrazyl radical (DPPH^•^) and compared to that of a standard (*i*.*e*., ascorbic acid). Microvolumes of methanolic stock solutions of the samples or the standard were added to 2 mL of a 125 μM of DPPH^•^ methanolic solution. The DPPH^•^ stock solutions were prepared daily and kept at 4°C until it was used. The absorbance at 515 nm was measured over time (every 30 second) on a Varian Cary 50 UV-Vis. Spectrophotometer until the reaction time reached 16 minutes. The percentage of remaining DPPH^•^ as assessed by the absorbance at 515 nm was plotted to evaluate the EC_50_ (concentration of the antioxidant required to scavenge the initial DPPH^•^ concentration by 50%) [51–53]. Low EC_50_ values reflect high antioxidant capacities of the considered systems.

### Preparation of the Flavonoids and Metal Solutions for Biochemical Analyses

Solutions of flavonoids and metals were prepared immediately before use. Quercetin, (+)-catechin and rutin were first dissolved in DMSO and then diluted to a final DMSO concentration of 0.1% in the reaction tube. At this concentration, DMSO had no appreciable effect on RBC hemolysis. Flavonoids were used at two different final concentrations in the reaction tube (200 μM and 100 μM). Fe, Cu and Zn stock solutions were first prepared in distilled water, and then diluted to a final concentration of 400 μM in the reaction tubes.

### Ethics Statement

This study was carried out following the Algerian law (25/2006, Resolution No. 387). The donor gave his written informed consent and the ethic committee at the University of Tlemcen approved the study.

### Preparation of Human Blood Samples

Human RBCs were separated from heparinized blood that was drawn from a healthy donor. The blood was centrifuged at 2000 rpm for 10 min to separate the RBCs from plasma, and then the RBCs were washed three times with phosphate-buffered saline solution (PBS, pH 7.4) until the color of the supernatant turned clear [54].

### Hemolysis Assay

The 5% suspension of RBCs in PBS (pH 7.4) was incubated under air atmosphere at 37°C for 30 minutes with or without the flavonoid, into which an aqueous solution of the metals was added to initiate hemolysis. The reaction mixture was shaken gently while being incubated at 37°C. The extent of hemolysis was determined spectrophotometrically as described elsewhere [55]. Briefly, aliquots of the reaction mixture were taken out after 4 hours of incubation, diluted with PBS, and centrifuged at 2000 rpm for 10 min to separate the RBCs. The percentage of hemolysis was determined by measuring the absorbance of the supernatant at 540 nm and compared with that of complete hemolysis by treating the same RBC suspension with distilled water.

$${\%}_{hemoysis}=(\frac{A_{sample}}{A_{total}})\times 100$$

Where: A_sample_ is the absorbance of the test sample collected 4 hours from the reaction mixture, and A_total_ is the total hemoglobin content of the cells.

### Determination of Markers of the Oxidant/Antioxidant Status

Hemolysate reduced glutathione (GSH) levels were assayed by a colorimetric method of Ellman [56] in which the reduction of Ellman’s reagent (5,5-dithiobis-(2-nitrobenzoic) acid DTNB) by thiol groups of GSH to generate 2-nitro-5-thiobenzoic acid which has yellow colour, according to a Sigma Aldrich Kit (Saint Louis, MO, USA). The absorbance at 412 nm was measured, and the GSH concentration was then determined with the GSH standard curve.

The catalase activity (CAT, EC 1.11.1.6) was measured by spectrophotometric analysis of the decomposition rate of hydrogen peroxide according to the method of Aebi [57]. The results were expressed as unit of catalase per mg of Hg.

Carbonyl proteins (marker of proteins oxidation) were assayed by the derivatisation of carbonyl protein groups with 2,4-dinitrophenylhydrazine (DNPH) leading to the formation of stable dinitrophenyl (DNP) hydrazone adducts, which can be detected spectrophotometrically at 375 nm (Sigma Aldrich Kit Saint Louis, MO, USA). Oxidized BSA standard was used for the standard curve [58].

Malondialdehyde (MDA, marker of lipid peroxidation) was estimated by the method of Draper and Hadley et al. [59] using thiobarbituric acid (TBA). Absorbance was measured at 532 nm. The results were expressed as nmol per g of Hb of MDA, using the molar extinction coefficient of chromophore (1.56 × 10^5^ M^-1^ cm^-1^).

### Statistical Methods

The results are presented as means ± standard deviations of at least three repetitions. Significant difference s (P<0.05) or (P<0.01) were determined by paired Student t-test to compare means between controls and treated RBC’s. All tests were performed using STATISTICA-8 program (StatSoft, Tulsa, OK).

## Results

### Absorption and Stabilities of metal Complexes

In the human body, Fe(III) is mainly exposed to neutral pH (pH ~7.4). To maintain its solubility under physiological conditions, Fe is therefore constantly bound to proteins (hemoglobin, transferrin) and to low molecular weight moderate chelators such as citrates. The non-protein bounded iron is called "*labile iron pool*" or "*chelatable iron pool*", and could be the target of exogenous chelators such as polyphenols [60, 61]. Polyaminocarboxylate-type ligands such as citrate and nitrilotriacetic acid (NTA) are currently used in model systems to ensure Fe(III) solubility [62]. For instance, they have been used to study Fe(III) uptake processes by siderophores [63], polyphenols [64, 65], hydroxamic acids [66] or transferrin [67] at physiological pH. The aminotricarboxylate chelator NTA can be considered as a model of citric acid and is one of the most studied organic chelators. The speciation of its ferric complexes is well known [68, 69].

**Fig 3:**
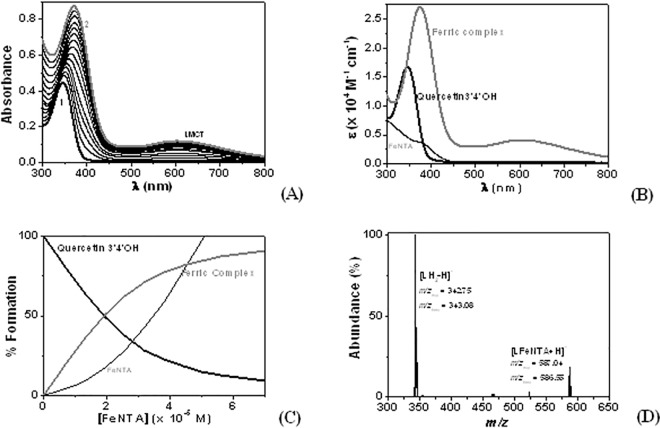
Absorption spectrophotometric titration of 3,5,7-tri-O-methyl-quercetin (quercetin3’4’OH). (A) Absorption spectra, (B) absorption electronic spectra, and (C) complex formation evolution as a function of the [Fe**NTA**]_0_ of the Fe**NTA** complex with quercetin3’4’OH. Solvent: CH_3_OH/H_2_O (80/20 by weight); pH = 7.4 (Hepes buffer); *T* = 25.0(2°C; *l* = 1 cm. [Quercetin 3’4’OH]_0_ = 2.89 × 10^−5^ M. (D) Electrospray mass spectra of quercetin3’4’OH ferric complex (noted LH_2_) in the presence of NTA. Solvent: CH_3_OH, capillary voltage = 4000 V. [L.Fe**NTA**]_tot_ = 5 × 10^−5^ M; Negative mode; Fragmentor = -100 V.

Absorption spectrophotometric titrations of quercetin and its synthetic methylated analogues (**Fig 2**) with Fe**NTA** were therefore undertook at pH 7.4 in CH_3_OH/H_2_O solvent in order to enlighten which chelation site is the favourable one (catecholate *versus* α-hydroxycarbonyl *versus* β-hydroxcarbonyl) and to compare their stability with the other flavones such as rutin (catecholate *versus* β-hydroxcarbonyl) or (+)-catechin (catecholate), two other widely distributed flavonoids in the plants kingdom. As exemplified in **Fig 3**, the absorption titration of the 3,5,7-tri-O-methyl-quercetin (quercetin3’4’OH) with Fe**NTA** revealed, in addition to the bathochromic shift of the π-π* transitions lying at ~ 350 nm, the formation of a broad absorption band (ligand-to-metal Charge Transfer LMCT) in the visible region that is characteristics of Fe(III) binding [70] by catecholate based systems.

These absorption transitions lying in the visible region can be indeed attributed to charge transfers from the p_π_ orbitals of catechol or α-hydroxy-carbonyl oxygens to the *d*_π_ and *d*_σ_ orbitals of iron(III) and are a clear spectrophotometric signature of the ferric metal complexes. The red shift of the π-π* transitions of the polyphenolic compounds can be ascribed to electronic effects induces by Fe(III) complexation. Similar effects are indeed observed upon deprotonation of the phenolic sites. LMCT transitions as well as bathochromic shifts of the flavonoids π-π* transitions were also observed for the other ferric-complexes with Fe**NTA** (**Table 1**). The absorption spectrophotometric data sets of quercetin and its methylated analogues, (+)-catechin and rutin have been statistically processed [48] to determine the successive stability constants (**Table 1**). Stoichiometric 1:1:1 Fe**NTA** complexes were systematically evidenced and confirmed the previous studies on catecholate [65] or hydroxamate [71] based systems with Fe**NTA**. The ternary complexes with Fe**NTA** were also further characterized by ESI-MS which reveals the presence of monocharged (positively or negatively) isotopic patterns that agree with the simulated data (*i*.*e*., Quercetin3’4’OH in **Fig 3**). Quercetin3’4’OH (**Fig 3**), quercetin, rutin and (+)catechin were shown to be the best Fe(III) chelators examined herein, suggesting that the catecholate is a more efficient binding site for Fe**NTA**. In the presence of Fe**NTA**, quercetin3OH and quercetin3,5OH were also shown to display LCMT transitions in the visible region (but lying at much higher energies), [72] which indicates that this molecule, through its α-hydroxycarbonyl unit, also binds Fe**NTA**. However, this binding site afforded less stable ferric complexes with respect to catecholate (quercetin3’4’OH, rutin or quercetin). Last but not the least, the absorption titration of the quercetin5OH with Fe**NTA** demonstrates that the β-hydroxcarbonyl, within this flavonol series, doesn’t bind Fe(III).

**Table 1:** Stability constants of the FeNTA, Cu and Zn complexes with rutin, (+)-catechin and quercetin and its O-methylated analogues^a^.

Flavonoid		Q	Q3OH	Q5OH	Q35OH	Q3'4'OH	(+)Catechin	Rutin
**Fe**	*K*_**L**.Fe**NTA**_ (σ) [M^-1^]	1.0(7) × 10^5^	2.5(1.1) × 10^4^	nc	6.3(8.7) × 10^4^	2.0(1.4) × 10^4^	1.6(1) × 10^6^^*b*^	2.0(9) × 10^5^
	*K*_**L**.Fe**NTA**_ (σ) [M^-1^]	620			425/515	600	570	598
	ε^λmax^ (10^3^ M^-1^ cm^-1^)	3.7	4.8		4.8/1.1	4.05	2.4	2.7
**Cu**	*K*_**L**.Cu_ (σ) [M^-1^]	1.0(5) × 10^6^	nd	nd	nd	nd	1.0(0.5) × 10^4^	4.0(9) × 10^4^
	*K*_**L**2.Cu_ (σ) [M^-1^]	6.3(4.4) × 10^4^					-	-
**Zn**	*K*_**L**.Zn_(σ) [M^-1^]	1.70(8) × 10^4^	nd	nd	nd	nd	~ 10^2^	4.2(3) × 10^2^

Q = quercetin, Q3’4’OH = Quercetin3’4’OH, Q35OH = Quercetin35OH, Q3OH = Quercetin3OH, Q5OH = Quercetin5OH. log *K*_Fe**NTA**_ = 6.0 at pH = 7.4.

^*a*^ Solvent: CH_3_OH/H_2_O (80/20 by weight); pH = 7.4 (Hepes buffer); *I* = 0.1 M (Hepes); *T* = 25.0(2°C.

^*b*^ Solvent: H_2_O; pH = 7.4 (Hepes buffer 0.05 M); *T* = 25.0(2°C;. nd: not determined; nc: no to weak complexation. σ = standard deviation.

This absorption spectrophotometric investigation of this flavonoids series thus enables us to establish the binding sequence with respect to Fe(III): quercetin3'4'OH > quercetin3OH >> quercetin5OH. The LMCT absorption band measured for quercetin 3'4'OH was found to be centred at about 600 nm as observed for the quercetin LMCT absorption band (λ_max_ LCMT = 608 nm). Assuming these facts, we can propose that the complexation of the quercetin first proceeds through its catecholate binding site.

The Cu(II) and Zn(II) complexation properties of quercetin, rutin and (+)catechin were then examined using the same analytical approach. The stability sequence satisfactorily follows the Irving-Williams order [73] with the Cu(II) complexes being the more stable with respect to Zn(II). In addition, cupric monochelate LCu and bischelates L_2_Cu were characterized for quercetin in agreement with reported data [74].

### Antioxidant Properties

**Table 2**summarizes the results of the antiradical activities obtained using the 2,2-diphenyl-1-picrylhydrazyl (DPPH) radical scavenging assay.

**Table 2:** Effective concentration (EC_50_±SD) of the investigated flavonoids and standard (*i*.*e*., ascorbic acid). SD: Standard Deviation.

Compound	EC_50_(μM)	Slope/R^2^
Quercetin	12.02±1.08	7.67/0.998
Rutin	12.62±0.78	7.13/0.999
Quercetin3’,4’OH	18.16±0.65	6.61/0.998
catechin	21.11±0.86	4.62/0.986
***Ascorbic acid***	***24*.*84***±0**.**12	**3.96/*0*.*998***
Quercetin3OH	25.72±0.46	3.24/0.999
Quercetin3,5OH	37.86±0.22	2.11/0.999
Quercetin5OH	50.27±0.08	1.79/0.999

**Fig 4:**
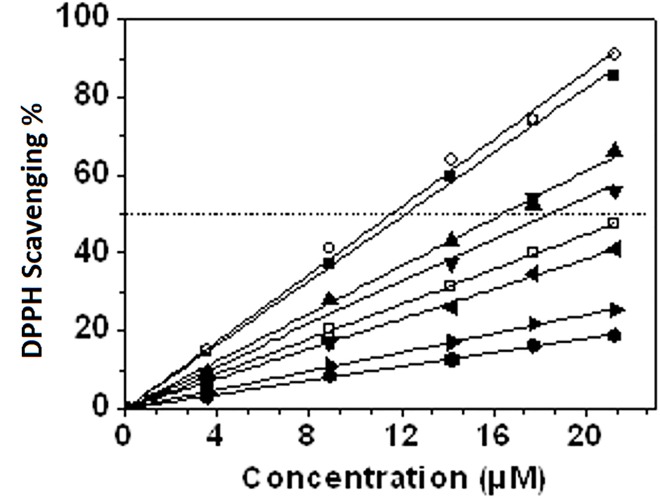
DPPH scavenging activity of studied flavonoids and the standard: ○-Quercetin, ■-Rutin, ▲-Quercetin 3’,4’OH,▼-Catechin, □-Ascorbic acid, ◄-Quercetin 3 OH, ►-Quercetin 3,5 OH, ●-Quercetin 5 OH. The average error on the inhibition percentage was estimated to be 4% for all the examined concentrations.

Ascorbic acid was used for comparisons purposes. All the compounds investigated in this work exhibited potent antiradical activities. The values of the EC_50_ (effective concentration of the antioxidant by which the initial DPPH concentration is decreased by 50%) are inversely proportional to the antioxidant activity (*i*.*e*., lower the EC50 is, higher the antioxidant activity of a compound is). Among the tested compounds, quercetin (12.02 ± 1.08 μM) and rutin (12.62 ± 0.78 μM) exhibited the highest radical scavenging activity (**Fig 4**). It was found to be two times more efficient as the standard ascorbic acid. Generally speaking, compounds bearing a catechol B subunit (quercetin, rutin, (+)catechin and quercetin3’4’OH) were found to be more efficient than ascorbic acid. The antiradical activity sequence was established as follows: quercetin > rutin > quercetin3’4’OH > (+)catechin > ascorbic acid > quercetin3OH > quercetin3,5OH > quercetin5OH and perfectly matches with the one established for Fe(III) binding. It thus demonstrates that, within the flavonoids sub-family, an efficient antiradical/antioxidant compound is also an efficient Fe(III) chelator.

### *In vitro* Activities

**Fig 5:**
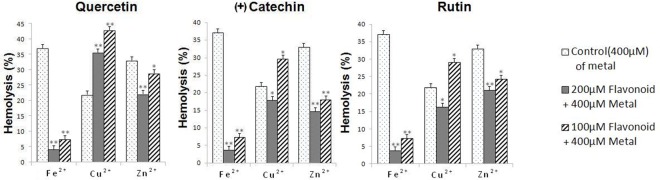
Hemolysis of a 5% human RBCs treated with metal ions (400 μM) in the absence or the presence of flavonoids (200 μM and 100µM) under air atmosphere at 37°C. The significance of the differences between treated RBCs and control was determined by the Student t-test: *P < 0.05. **P < 0.01.

We then investigated the effect of metal addition on the hemolysis of RBC membranes (**Fig 5**). For the sake of solubility, Fe was added as its ferrous state. It is anticipated that Fe(II) will be readily oxidized to the Fe(III) state [75]. Addition of 400 μM of Fe^2+^, Zn^2+^ and Cu^2+^ led to 37%, 22% and 32% of RBC hemolysis, respectively.

We have shown above that quercetin, rutin and (+)catechin, that display a B-catecholate subunit are strong radical scavengers and good Fe(III) chelators. It is therefore not surprising to observe that these three natural flavonoids are able due to their intrinsic properties to markedly prevent RBC hemolysis in the presence of Fe overload. Quercetin displays potent RBC anti-hemolytic activity and decreases the hemolysis percentage induced by metal supply (37% at 400 μM Fe^2+^) to very low levels (4.18% and 7.45% at 200 and 100 μM of quercetin, respectively,). However, quercetin did not show the same extent of protection in the presence of Zn^2+^ ions. It decreases the initial hemolysis from 33% (400 μM of Zn^2+^) to only 28% and 22% at 100 μM and 200 μM of quercetin, respectively. This is in agreement with the weaker chelating capacities of catecholate-based ligands toward Zn^2+^ (**Table 1**). Strikingly, quercetin used at both concentrations (100 μM and 200 μM) shows a prooxidant activity in the presence of Cu^2+^. RBC Hemolysis increased from 22% (400 μM of Cu^2+^) to 35% (200 μM) and 42% (100 μM), respectively. These results are consistent with previous works that demonstrate Cu(II) reduction by catechols to more toxic Cu(I) ions.

(+)Catechin and rutin, both displaying a B-catecholate subunit, led to comparable results. Their firm Fe binding capacities significantly reduce the RBC hemolysis induced by Fe^2+^ supply. Rutin showed the same anti-hemolytic activities with Zn^2+^ than quercetin, while catechin displayed a higher anti-hemolytic effect in the presence of Zn^2+^. In the presence of Cu^2+^, catechin and rutin had a weak protective effect at 200 μM, whereas this moderate protective effect turns to a prooxidant effect at 100 μM raising the RBC hemolysis percentage to ~ 29% for both catechin and rutin.

**Fig 6:**
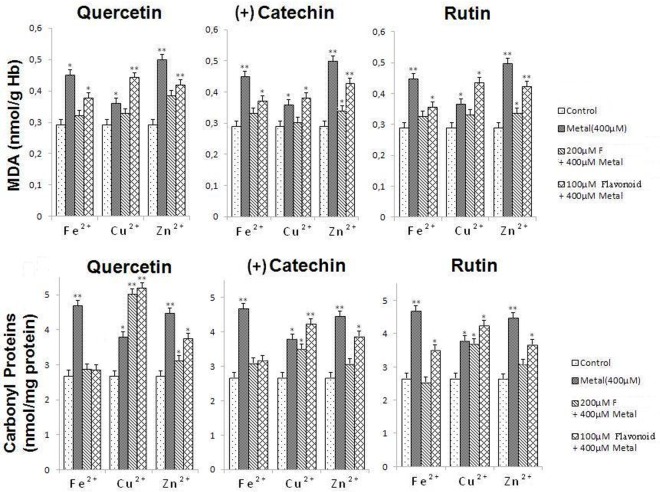
Oxidant status of a 5% human RBCs treated with metal ions (400 μM) in the absence or the presence of flavonoids (200 μM and 100µM) under air atmosphere at 37°C. The significance of the differences between treated RBCs and control was determined by the Student t-test: *P < 0.05. **P < 0.01.

We then turned our attention to the oxidant status (*i*.*e*., by measuring carbonyl proteins and MDA) of the treated RBCs under different experimental conditions (**Fig 6**).

Levels of carbonyl proteins were raised from 2.66 nmol/mg (control) to 4.67 (Fe^2+^), 3.78 (Cu^2+^) and 4.46 (Zn^2+^) nmol/mg of proteins, respectively, which confirmed the prooxidant effect of the metal ions**.** In the presence of Fe^2+^, the three flavonoids turned back the carbonyl proteins to their normal levels (*i*.*e*., quercetin is the most potent). When treated with Zn^2+^ ions, carbonyl proteins levels of the treated RBCs also decreased for the three flavonoids; nonetheless this effect was found to be weaker by comparison with Fe^2+^. For the RBCs treated with Cu^2+^, the carbonyl proteins levels further increased whatever the flavonoid considered (100 μM) and exemplify the prooxidant properties of the cuprous catecholate complexes.

With respect to another oxidant marker such as MDA (**Fig 6**), addition of excess of Fe^2+^ and Zn^2+^ to 5% human RBCs strongly raised its levels from 0.29 nmol/g Hb (control) to 0.44 (Fe^2+^) and 0.49 nmol/g Hb (Zn^2+^), respectively. With Cu^2+^, this effect was much weaker (increase from 0.29 nmol/g Hb for the control to 0.35 nmol/g Hb for 400 μM Cu^2+^). The antioxidant and chelating properties of the three flavonoids are clearly in agreement with the decrease of the MDA marker that was induced by Fe^2+^ and Zn^2+^ overload. By contrast, formation of the prooxidant Cu(I) complexes with catecholate-based flavonoids (100 μM, see above) significantly increases the MDA concentrations and reflects altered oxidant status. The use of higher concentrations of flavonoids (200 μM) slightly reverses this deleterious state.

**Fig 7:**
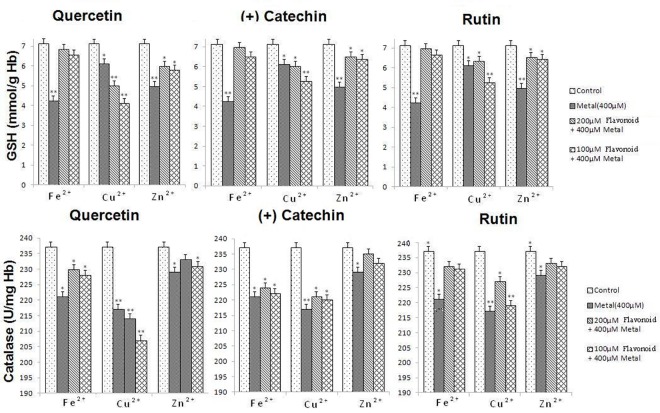
Antioxidant status of a 5% human RBCs treated with metal ions (400 μM) in the absence or the presence of flavonoids (200 μM and 100 μM) under air atmosphere at 37°C. The significance of the differences between treated RBCs and control was determined by the Student t-test: *P < 0.05. **P < 0.01.

With respect to the antioxidant markers (GSH and catalase), the glutathione GSH levels (7.12 mmol/g Hb for the control) decreased for all the metals considered. This decrease is marked in the case of Fe^2+^ and Zn^2+^ (4.25 and 4.97 mmol/g Hb, respectively) in agreement with the RBCs hemolysis and the markers of the oxidant status. As seen previously, the GSH levels also met the levels of the controls when the RBCs supplied with Fe^2+^ were treated with the three flavonoids. The same situation stands for Zn^2+^ but the GSH levels were not restored as observed for Fe^2+^. Once again, a clear prooxidant effect can be seen when the Cu^2+^ treated RBCS were supplied with 100 μM of the flavonoids (**Fig 7**).

As for GSH, catalase levels (237 U/mg Hb for the control) significantly decreased for the redox active metal ions Fe^2+^ and Cu^2+^, confirming that the antioxidant status of the RBCs was considerably altered. The effect is less obvious for Zn^2+^. As seen previously, supplying flavonoids to the treated RBCs increases the catalase levels in particular in the case of Fe^2+^, the marked effect being obtained for rutin. By contrast with the other (anti)oxidant markers, the catalase activity of the Cu^2+^-treated RBCs was almost not affected in the presence of catechin and increases for rutin. However, in the presence of quercetin (100 μM) and Cu^2+^, the catalase activity significantly fell off. The catalase activity of the Zn^2+^-treated RBCs does not seem to change significantly under the effect of flavonoids even at concentrations of 200 μM.

## Discussion

In this study, red blood cells (RBCs) were subjected to *in vitro* extracellular metal ion-induced hemolysis. We investigated the response of RBCs to the generated damage and their (anti)oxidant status. The impact of three relevant flavonoids was examined on the hemolysis and the (anti)oxidant markers of the treated RBCs. These biochemical data were compared to physico-chemical data related on metal binding.

In human cells, H_2_O_2_, a by-product of oxygen metabolism, may be present at micromolar concentrations and is relatively harmless as it reacts with biomolecules at relatively low rates. In addition, specific enzymes facilitate its removal. However, metal (*e*.*g*., Fe, Cu) ions can catalyze the conversion of H_2_O_2_ into a highly reactive and damaging hydroxyl radical (HO^•^). Fe mostly occurs in its +II and +III oxidation states. The ferrous ions are soluble in biological fluids and generate in the presence of oxygen or hydrogen peroxide (*i*.*e*., Fenton reaction) damaging and deleterious reactive oxygen species (ROS). The ferrous ions are unstable in aqueous media and tend to rapidly react with molecular oxygen to form ferric ions and superoxide anion radical [76]. On the other hand, polyphenols can either bind Fe(II), thus preventing this pro-oxidant cation from reacting with hydrogen peroxide or promote the oxidation of the less stable ferrous complexes into more stable ferric ones (*i*.*e*., auto-oxidation) that cannot participate any longer to the Fenton reaction. Our findings support previous studies [77] that showed that flavonoids act as valuable antioxidants because of their chelating properties. It was also reported that metal chelates are more prone to scavenge free radicals than the free flavonoids.

Exposure of RBCs to the Fe-induced stress leads to intracellular damages in the form of hemoglobin oxidation and membrane-bound hemichromes production and modification of the membrane components as a consequence of enhanced lipid peroxidation [78]. This damage led to important hemolysis (37% at 400 μM under our experimental conditions). Pre-treatment of RBCs with quercetin, rutin and catechin almost completely prevented the hemolysis caused by Fe^2+^. This can be explained by several closely related events. These three relevant flavonoids act as efficient oxygen radical scavengers as shown by their potent antiradical properties towards the DDPH^•^ radical assay (**Table 2**) [79]. The formation of very stable Fe complexes (**Table 1**) also prevents ROS formation (*e*.*g*., it has been shown that only traces of H_2_O_2_ are detected in presence of Fe ions and quercetin [80, 81]. In addition, Fe(II) is rapidly oxidised to Fe(III) in the presence or absence of the polyphenol and the oxidation rate constants of the ferrous complexes have been demonstrated to be intimately related to the anti-oxidant properties of the flavonoid compounds [82].

These features are reflected by the oxidant and antioxidant biomarkers. Both oxidant markers—MDA and carbonyl proteins—significantly increased (**Fig 6**) on supply of the RBCs with Fe^2+^ cations in line with an Fe-induced oxidative stress of the RBCs that ultimately leads to hemolysis. However, pre-treatment of the RBCs with quercetin, rutin or (+)catechin maintained the MDA and carbonyl proteins at initial levels. On the other hand, the antioxidant markers—GSH and catalase activity—(**Fig 7**) also significantly decreased in the presence of 400 μM of Fe^2+^ ions in agreement with the oxidative stress experienced by the cells. Following a pre-treatment of the RBCs with quercetin, rutin or (+)catechin in the presence of 400 μM of Fe^2+^, the GSH concentrations were found to be at normal levels even at low flavonoid concentration (100 μM). The catalase activity was also reinstated at initial levels for quercetin and rutin pre-treatments. By contrast, the catalase activity for the RBCs pre-treated with (+)catechin was comparable to that of 400 μM Fe^2+^ alone. Altogether, these data clearly demonstrated that the antioxidant/chelating properties of the catecholate-based flavonoids prevented Fe^2+^ toxicity thus allowing regulating the redox homeostasis of the treated RBCs.

Cu(II) stands in contrast with Fe(II). Reaction of free Cu(II) with H_2_O_2_ can occur via a “free radical” mechanism in which Cu(II) oxidizes H_2_O_2_ to O_2_^•-^ with Cu(I) being formed. Cu(I) which is produced can react with excess of H_2_O_2_ to form HO^•^ (Fenton-like reaction). It was also proposed that Cu(I) (generated from the reaction between Cu(II) and O_2_^•−^) reacted with H_2_O_2_ to lead to a deleterious and highly reactive Cu(III) intermediate oxidizing species [30] Cu can also induce oxidative stress and subsequent damages to cells (by alteration of membrane permeability or by affecting chromatin structure, protein synthesis, and various enzyme activities) through redox cycling between Cu(I) and Cu(II), particularly in the presence of H_2_O_2_ (*i*.*e*., Fenton-like reaction), a by-product of oxygen metabolism [83, 84]. Cu was also shown to decrease the glutathione levels [85] as also evidenced in the present study (**Fig 7**). We demonstrated that Cu^2+^ ions led to less harmful damages (22% of hemolysis) than Fe^2+^ does (37% of hemolysis). Similarly to Fe^2+^ (see above), the higher levels of carbonyl proteins and MDA, used as oxidant markers (**Fig 6**), confirmed that the RBCs hemolysis mainly resulted from metal-induced oxidative stress. Carbonyl proteins levels seem to be, however, more altered than MDA. By contrast with Fe^2+^, complexation of Cu^2+^ by catecholate based ligands such as particular flavonoids (*i*.*e*., those bearing catechol binding sites) do not represent a significant advantage for the antioxidant activity as catechol-mediated reduction to Cu(I) can occur and trigger and exacerbate the copper toxicity [86]. Pre-treatment of the RBCs with the investigated flavonoids indeed clearly demonstrated a prooxidant effect as evidenced by the further increase of the hemolysis to 42%, 30% and 29% for quercetin, (+)catechin and rutin, respectively. We demonstrated that the investigated flavonoids are able to complex Cu^2+^ (**Table 1**), but led to less stable complexes than with Fe^3+^. The Cu-quercetin complexation was suggested to occur *via* the 4-keto group of the C-ring with additional involvement of the 3OH or 5OH group [74]. Complexation of Cu(II) by catecholate-based ligands was demonstrated to favour metal reduction and lead to more deleterious Cu(I) cations [86]. For instance, it was shown that during the Cu-initiated autoxidation of quercetin, H_2_O_2_ rapidly accumulates. Furthermore, the main autoxidation products of quercetin was shown to be the solvent adducts on the p-quinonemethide intermediate formed upon two-electron oxidation of quercetin [80]. For 100 μM quercetin, the oxidant markers (**Fig 6**) were significantly increased while the antioxidant ones (**Fig 7**) concomitantly decreased in agreement with an altered oxidative environment induced by Cu^2+^ complexation. Increasing quercetin concentration up to 200 μM improved the redox status of the treated RBCs. Even though the same effects were also observed for rutin and (+)catechin, the magnitude of oxidative stress was not significant as for quercetin. This can be related to the nature of the Cu complexes with these two polyphenols. Rutin is substituted on its 3OH position by a rutinoside while (+)catechin is a flavan-3-ol. At high concentrations (200 μM), an antioxidant activity was observed for (+)catechin and rutin, while they displayed a prooxidant effect at much lower concentrations (100µM). It was proposed that for polyphenols exhibiting both antioxidant and prooxidant activity, a redox-cycling pathway may occur at low concentrations when there is not enough polyphenols to scavenge radicals, and at higher concentrations radicals are scavenged at once [87].

A particular behaviour among the investigated metals is occupied by the redox inert metal ion Zn^2+^. Zn^2+^ is an essential component of numerous proteins involved in the defence against oxidative stress [88]. It is the most common non-redox transition metal [89] and its total concentration in human plasma is ranging between 12 and 20 mM [90]. However, its free concentration is several orders of magnitude lower, mainly due to binding by albumin [91, 92]. Cellular Zn homeostasis is tightly regulated because of the regulatory roles of intracellular Zn^2+^. Specialized proteins are responsible for controlling Zn import and export, as well as its intracellular distribution [93]. We have showed that a supply of Zn^2+^ cations at a concentration of 400 μM is responsible of potent hemolysis of RBCs (32%,). At such high concentrations, Zn^2+^ could also affect transport systems across the RBCs and therefore increase the permeability of the membranes to small molecules and lead to subsequent hemolysis [94]. Flavonoids protection was clearly shown to be less efficient against the Zn^2+^-induced hemolysis (16% of the initial hemolysis for quercetin and rutin and 55% for catechin at a concentration of 200 μM,). This is most likely due to the less stable complexes that can be formed with the three flavonoids considered in this work. Even though Zn^2+^ is an inert metal cation, it induces a significant increase of the oxidant markers such as MDA and carbonyl proteins (**Fig 6**). Flavonoids protection was efficient at a concentration of 200 μM. The GSH levels were highly decreased by Zn^2+^ ions, but pre-incubation with flavonoids kept these values close to their normal levels (**Fig 7**). The catalase activity was slightly affected by Zn^2+^ and pre-incubation with flavonoids allowed maintaining the catalase to its normal levels. We thus hypothesized that flavonoids mainly act as antioxidant compounds rather than binding the supplied Zn^2+^.

## Conclusion

In summary, flavonoids possessing a catecholate group are more likely to interact with metals especially Fe. Our results showed that at high Fe and Zn concentrations, flavonoids were able to inhibit their hemolytic activity. However, when interacting with Cu a weak antioxidant effect was observed at high metal concentrations, and prooxidant activity was observed at low concentrations. This was corroborated by the data obtained with oxidative stress markers. Flavonoids are then able to exhibit both prooxidant and antioxidant activities depending on their concentration and on the metal concentration.

## Supporting Information

S1 Fig Absorption spectrophotometric titration of quercetin by FeNTA. (A) Absorption spectra, (B) absorption electronic spectra, and (C) complex formation evolution as a function of the [Fe**NTA**]_0_. Solvent: CH_3_OH/H_2_O (80/20 by weight); pH = 7.4 (Hepes buffer); *T* = 25.0(2°C; *l* = 1 cm; (1) [Quercetin]_0_ = 1.90 × 10^−5^ M; (2) [Fe**NTA**]_0_/[Quercetin]_0_ = 5.58. (D) Electrospray mass spectra of quercetin (noted LH_5_) ferric complex in the presence of NTA. Solvent: CH_3_OH, capillary voltage = 4000 V. [LH_3_Fe**NTA**]_0_ = 5 × 10^−5^ M; negative mode; Fragmentor = -200 V.
(DOCX)

S2 Fig Absorption spectrophotometric titration of rutin by FeNTA. (A) Absorption spectra, (B) absorption electronic spectra, and (C) complex formation evolution as a function of the [Fe**NTA**]_0_. Solvent: CH_3_OH/H_2_O (80/20 by weight); pH = 7.4 (Hepes buffer); *T* = 25.0(2°C; *l* = 1 cm. (1) [Rutin]_0_ = 4.94× 10^−5^ M; (2) [Fe**NTA**]_0_/[Rutin]_0_ = 2.02. (D) Electrospray mass spectra of rutin (noted LH_4_) ferric complex in the presence of NTA. Solvent: CH_3_OH, capillary voltage = 4000 V. [LH_2_Fe**NTA**]_0_ = 5 × 10^−5^ M; positive mode; Fragmentor = +50 V.
(DOCX)

S3 Fig Absorption spectrophotometric titration of quercetin3OH by FeNTA. (A) Absorption spectra, (B) absorption electronic spectra, and (C) complex formation evolution as a function of the [Fe**NTA**]_0_. Solvent: CH_3_OH/H_2_O (80/20 by weight); pH = 7.4 (Hepes buffer); *T* = 25.0(2°C; *l* = 1 cm. (1) [Quercetin3OH]_0_ = 1.51 × 10^−5^ M; (2) [Fe**NTA**]_0_/[Quercetin3OH]_0_ = 2.49. (D) Electrospray mass spectra of quercetin3OH (noted LH) ferric complex in the presence of NTA. Solvent: CH_3_OH, capillary voltage = 4000 V. [LFe**NTA**]_0_ = 5 × 10^−5^ M; positive mode; Fragmentor = +150 V.
(DOCX)

S4 Fig Absorption spectrophotometric titration of quercetin35OH by FeNTA. (A) Absorption spectra, (B) absorption electronic spectra, and (C) complex formation evolution as a function of the [Fe**NTA**]_0_. Solvent: CH_3_OH/H_2_O (80/20 by weight); pH = 7.4 (Hepes buffer); *T* = 25.0(2°C; *l* = 1 cm; (1) [Quercetin35OH]_0_ = 4.0 × 10^−5^ M; (2) [Fe**NTA**]_0_/[Quercetin35OH]_0_ = 1.77.
(DOCX)

S5 Fig Absorption spectrophotometric titration of quercetin by Cu(II). (A) Absorption spectra, (B) absorption electronic spectra, and (C) complex formation evolution as a function of the [Cu(II)]_0_. Solvent: CH_3_OH/H_2_O (80/20 by weight); pH = 7.4 (Hepes buffer); *T* = 25.0(2°C; *l* = 1 cm. (1) [Quercetin]_0_ = 4.98 × 10^−5^ M; (2) [Cu(II)]_0_/[Quercetin]_0_ = 0.94.
(DOCX)

S6 Fig Absorption spectrophotometric titration of quercetin by Zn(II). (A) Absorption spectra, (B) absorption electronic spectra, and (C) complex formation evolution as a function of the [Zn(II)]_0_. Solvent: CH_3_OH/H_2_O (80/20 by weight); pH = 7.4 (Hepes buffer); *T* = 25.0(2°C; *l* = 1 cm. (1) [Quercetin]_0_ = 4.94 × 10^−5^ M; (2) [Zn(II)]_0_/[Quercetin]_0_ = 1.95.
(DOCX)

S7 Fig Absorption spectrophotometric titration of rutin by Cu(II). (A) Absorption spectra, (B) absorption electronic spectra, and (C) complex formation evolution as a function of the [Cu(II)]_0_. Solvent: CH_3_OH/H_2_O (80/20 by weight); pH = 7.4 (Hepes buffer); *T* = 25.0(2°C; *l* = 1 cm. (1) [Rutin]_0_ = 4.94 × 10^−5^ M; (2) [Cu(II)]_0_/[Rutin]_0_ = 1.40. (D) Electrospray mass spectra of the rutin (noted LH_4_) Cu(II) complex. Solvent: CH_3_OH/H_2_O (80/20 *w/w*), capillary voltage = 4000 V. [LH_4_]_0_ = [Cu]_0_ = 8.7 × 10^−5^ M; negative mode; Fragmentor = -280 V.
(DOCX)

S8 Fig Absorption spectrophotometric titration of rutin by Zn(II). (A) Absorption spectra, (B) absorption electronic spectra, and (C) complex formation evolution as a function of the [Zn(II)]_0_. Solvent: CH_3_OH/H_2_O (80/20 by weight); pH = 7.4 (Hepes buffer); *T* = 25.0(2°C; *l* = 1 cm. (1) [Rutin]_0_ = 4.94 × 10^−5^ M; (2) [Zn(II)]_0_/[Rutin]_0_ = 28.54.
(DOCX)

S9 Fig Absorption spectrophotometric titration of (+)catechin by Cu(II). (A) Absorption spectra, (B) absorption electronic spectra, and (C) complex formation evolution as a function of the [Cu(II)]_0_. Solvent: CH_3_OH/H_2_O (80/20 by weight); pH = 7.4 (Hepes buffer); *T* = 25.0(2°C; *l* = 1 cm. (1) [(+)Catechin]_0_ = 2.55 × 10^−4^ M; (2) [Cu(II)]_0_/[(+)Catechin]_0_ = 1.50.
(DOCX)
