# Supplementary material for: In Vitro Antioxidant versus Metal Ion Chelating Properties of Flavonoids: A Structure-Activity Investigation
Source: PLoS One. 2016 Oct 27;11(10):e0165575. doi: 10.1371/journal.pone.0165575 (PMC5082868; doi:10.1371/journal.pone.0165575)
Supplement: S2 Fig — (A) Absorption spectra, (B) absorption electronic spectra, and (C) complex formation evolution as a function of the [FeNTA]0. Solvent: CH3OH/H2O (80/20 by weight); pH = 7.4 (Hepes buffer); T = 25.0(2°C; l = 1 cm. (1) [Rutin]0 = 4.94× 10^−5^ M; (2) [FeNTA]0/[Rutin]0 = 2.02. (D) Electrospray mass spectra of rutin (noted LH4) ferric complex in the presence of NTA. Solvent: CH3OH, capillary voltage = 4000 V. [LH2FeNTA]0 = 5 × 10^−5^ M; positive mode; Fragmentor = +50 V. (DOCX) [file pone.0165575.s002.docx]

(A) (B)

(C)(D)

**S2 Fig.** Absorption spectrophotometric titration of rutin by Fe**NTA**. (A) Absorption spectra, (B) absorption electronic spectra, and (C) complex formation evolution as a function of the [Fe**NTA**]_0_. Solvent: CH_3_OH/H_2_O (80/20 by weight); pH = 7.4 (Hepes buffer); *T* = 25.0(2) °C; *l* = 1 cm. (1) [Rutin]_0_ = 4.94× 10^-5^ M; (2) [Fe**NTA**]_0_/[Rutin]_0_ = 2.02. (D) Electrospray mass spectra of rutin (noted LH_4_) ferric complex in the presence of NTA. Solvent: CH_3_OH, capillary voltage = 4000 V. [LH_2_Fe**NTA**]_0_ = 5 × 10^-5^ M; positive mode; Fragmentor = +50 V.
