# Supplementary material for: In Vitro Antioxidant versus Metal Ion Chelating Properties of Flavonoids: A Structure-Activity Investigation
Source: PLoS One. 2016 Oct 27;11(10):e0165575. doi: 10.1371/journal.pone.0165575 (PMC5082868; doi:10.1371/journal.pone.0165575)
Supplement: S4 Fig — (A) Absorption spectra, (B) absorption electronic spectra, and (C) complex formation evolution as a function of the [FeNTA]0. Solvent: CH3OH/H2O (80/20 by weight); pH = 7.4 (Hepes buffer); T = 25.0(2°C; l = 1 cm; (1) [Quercetin35OH]0 = 4.0 × 10^−5^ M; (2) [FeNTA]0/[Quercetin35OH]0 = 1.77. (DOCX) [file pone.0165575.s004.docx]

 (A) (B)

(C)

**S4 Fig.** Absorption spectrophotometric titration of quercetin35OH by Fe**NTA**. (A) Absorption spectra, (B) absorption electronic spectra, and (C) complex formation evolution as a function of the [Fe**NTA**]_0_. Solvent: CH_3_OH/H_2_O (80/20 by weight); pH = 7.4 (Hepes buffer); *T* = 25.0(2) °C; *l* = 1 cm; (1) [Quercetin35OH]_0_ = 4.0 × 10^-5^ M; (2) [Fe**NTA**]_0_/[Quercetin35OH]_0_ = 1.77.
