# Supplementary material for: In Vitro Antioxidant versus Metal Ion Chelating Properties of Flavonoids: A Structure-Activity Investigation
Source: PLoS One. 2016 Oct 27;11(10):e0165575. doi: 10.1371/journal.pone.0165575 (PMC5082868; doi:10.1371/journal.pone.0165575)
Supplement: S5 Fig — (A) Absorption spectra, (B) absorption electronic spectra, and (C) complex formation evolution as a function of the [Cu(II)]0. Solvent: CH3OH/H2O (80/20 by weight); pH = 7.4 (Hepes buffer); T = 25.0(2°C; l = 1 cm. (1) [Quercetin]0 = 4.98 × 10^−5^ M; (2) [Cu(II)]0/[Quercetin]0 = 0.94. (DOCX) [file pone.0165575.s005.docx]

(A) (B)

(C)

**S5 Fig.** Absorption spectrophotometric titration of quercetin by Cu(II). (A) Absorption spectra, (B) absorption electronic spectra, and (C) complex formation evolution as a function of the [Cu(II)]_0_. Solvent: CH_3_OH/H_2_O (80/20 by weight); pH = 7.4 (Hepes buffer); *T* = 25.0(2) °C; *l* = 1 cm. (1) [Quercetin]_0_ = 4.98 × 10^-5^ M; (2) [Cu(II)]_0_/[Quercetin]_0_ = 0.94.
