# Supplementary material for: In Vitro Antioxidant versus Metal Ion Chelating Properties of Flavonoids: A Structure-Activity Investigation
Source: PLoS One. 2016 Oct 27;11(10):e0165575. doi: 10.1371/journal.pone.0165575 (PMC5082868; doi:10.1371/journal.pone.0165575)
Supplement: S7 Fig — (A) Absorption spectra, (B) absorption electronic spectra, and (C) complex formation evolution as a function of the [Cu(II)]0. Solvent: CH3OH/H2O (80/20 by weight); pH = 7.4 (Hepes buffer); T = 25.0(2°C; l = 1 cm. (1) [Rutin]0 = 4.94 × 10^−5^ M; (2) [Cu(II)]0/[Rutin]0 = 1.40. (D) Electrospray mass spectra of the rutin (noted LH4) Cu(II) complex. Solvent: CH3OH/H2O (80/20 w/w), capillary voltage = 4000 V. [LH4]0 = [Cu]0 = 8.7 × 10^−5^ M; negative mode; Fragmentor = -280 V. (DOCX) [file pone.0165575.s007.docx]

(A) (B)

(C)

**S7 Fig.** Absorption spectrophotometric titration of rutin by Cu(II). (A) Absorption spectra, (B) absorption electronic spectra, and (C) complex formation evolution as a function of the [Cu(II)]_0_. Solvent: CH_3_OH/H_2_O (80/20 by weight); pH = 7.4 (Hepes buffer); *T* = 25.0(2) °C; *l* = 1 cm. (1) [Rutin]_0_ = 4.94 × 10^-5^ M; (2) [Cu(II)]_0_/[Rutin]_0_ = 1.40. (D) Electrospray mass spectra of the rutin (noted LH_4_) Cu(II) complex. Solvent: CH_3_OH/H_2_O (80/20 *w/w*), capillary voltage = 4000 V. [LH_4_]_0_ = [Cu]_0_ = 8.7 × 10^-5^ M; negative mode; Fragmentor = -280 V.
