# Supplementary material for: In Vitro Antioxidant versus Metal Ion Chelating Properties of Flavonoids: A Structure-Activity Investigation
Source: PLoS One. 2016 Oct 27;11(10):e0165575. doi: 10.1371/journal.pone.0165575 (PMC5082868; doi:10.1371/journal.pone.0165575)
Supplement: S9 Fig — (A) Absorption spectra, (B) absorption electronic spectra, and (C) complex formation evolution as a function of the [Cu(II)]0. Solvent: CH3OH/H2O (80/20 by weight); pH = 7.4 (Hepes buffer); T = 25.0(2°C; l = 1 cm. (1) [(+)Catechin]0 = 2.55 × 10^−4^ M; (2) [Cu(II)]0/[(+)Catechin]0 = 1.50. (DOCX) [file pone.0165575.s009.docx]

(A) (B)

(C)

**S9 Fig.** Absorption spectrophotometric titration of (+)catechin by Cu(II). (A) Absorption spectra, (B) absorption electronic spectra, and (C) complex formation evolution as a function of the [Cu(II)]_0_. Solvent: CH_3_OH/H_2_O (80/20 by weight); pH = 7.4 (Hepes buffer); *T* = 25.0(2) °C; *l* = 1 cm. (1) [(+)Catechin]_0_ = 2.55 × 10^-4^ M; (2) [Cu(II)]_0_/[(+)Catechin]_0_ = 1.50.
